# Capsanthin-Loaded Micelles: Preparation, Characterization and *in vitro* Evaluation of Cytotoxicity Using MDA-MB-231 Breast Cancer Cell Line

**DOI:** 10.17113/ftb.60.03.22.7405

**Published:** 2022-09

**Authors:** Velmurugan Shanmugham, Ravi Subban

**Affiliations:** Department of Chemistry, Karpagam Academy of Higher Education, Salem-Kochi Highway, Eachanari, 641 021 Coimbatore, India

**Keywords:** capsanthin, breast cancer, MDA-MB-231 cell line, diosgenin polyethylene glycol succinate micelles, water solubility, bioavailability

## Abstract

**Research background:**

Breast cancer is one of the most common cancers and remains a major cause of morbidity and mortality among women worldwide. In developed countries, breast cancer as a multifactorial disease is a major health concern, and its incidence is constantly rising in low and middle-income countries. Numerous studies have demonstrated that phytochemicals such as carotenoids inhibit breast cancer growth and induce apoptosis. We recently enhanced the solubility of capsanthin in water by encapsulating it in diosgenin polyethylene glycol succinate, a novel non-ionic surfactant. Thus, this study aims to evaluate the cytotoxicity of water-soluble capsanthin-loaded micelles in MDA-MB-231 cells *in vitro* through tetrazolium dye MTT assay.

**Experimental approach:**

In the current study, capsanthin, a hydrophobic carotenoid, is extracted from sweet red pepper (*Capsicum annuum*). Capsanthin-loaded diosgenin polyethylene glycol succinate 1000 (cap-DPGS-1000) micelles were prepared from capsanthin extract (cap) and diosgenin polyethylene glycol succinate 1000 (DPGS-1000) using the solid dispersion method. The capsanthin extract and cap-DPGS-1000 micelles were characterized by UV-visible spectroscopy, high-performance liquid chromatography (HPLC), Fourier-transform infrared spectroscopy (FTIR), X-ray diffraction (XRD), particle size distribution, polydispersity, and scanning electron microscopy (SEM). The effects of capsanthin extract and cap-DPGS-1000 micelles on a human triple-negative breast cancer cell line (MDA-MB-231) were tested to check the cell viability, proliferation and cytotoxicity of the micelles.

**Results and conclusions:**

The solubility of encapsulated cap-DPGS-1000 micelles in water is greatly enhanced and leads to an increased scope for localized drug delivery, a better delivery option for treating residual cancerous tumours. The encapsulated capsanthin showed a sustained release in simulated intestinal fluid (pH=6.8). Our research proposes a sustained drug delivery system that ensures effective and controlled release to the affected site. The characterization data revealed no change in the structure and functional groups in the encapsulated capsanthin. The IC_50_ value of the cap-DPGS-1000 micelles against MDA-MB-231 breast cancer cells was (3.10±1.09) μg/mL, which is much lower than of capsanthin extract ((81.1±1.5) μg/mL). Capsanthin extract and capsanthin-loaded micelles are promising drug candidates to induce apoptosis and increase reactive oxygen species (ROS) in cancer cells.

**Novelty and scientific contribution:**

The result shows the cytotoxic effect of capsanthin and capsanthin-loaded micelles on MDA-MB-231 cell line for the first time. Capsanthin from sweet red pepper (*Capsicum annuum*) showed remarkable cytotoxic effect on the triple-negative MDA-MB-231 cell line.

## INTRODUCTION

Cancer, abnormal cell growth, can affect any organ or tissue of the body, and it is a multi-stage process that causes biological damage as a result of biochemical and molecular changes at the cellular level. The mortality rates depend on the prevention, early detection and effective treatment (*1*). An estimated 9.6 million cancer-related deaths occurred in 2018, as per the World Cancer Report (WHO) (*2*). Worldwide, breast cancer is still the principal cause of cancer mortality among women due to its destructive nature, metastasis and resistance to chemotherapeutic agents (*3*). Many studies have shown that the human diet plays a crucial role in preventing specific lifestyle diseases like cancer. Specifically, the Mediterranean diet, which consists of a large amount of fruit and vegetables, reduces the incidence of cancer (*4*).

Dietary carotenoids are potential antioxidants and offer numerous health benefits to the body. Specifically, they prevent certain cancer and age-related macular diseases. The most studied and reported carotenoids are lutein, zeaxanthin and lycopene (*5*). The red chili pepper (*Capsicum* species) is unique for its diversified carotenoid profile and composition (*6*). The intense red colour of chili is due to the presence of keto-carotenoids: capsanthin, capsorubin and capsanthin-5,6-epoxide, of which capsanthin is the major carotenoid (*7*). Capsanthin, a novel carotenoid, gained considerable research attention in the past owing to its usability as a natural food colourant. However, its high antioxidant and anticarcinogenic activities and potential use as a drug have been revealed recently (*8*). Studies have shown that capsanthin, a highly potent carotenoid has a significant anticancer effect and prevents and protects from the risk of cancer (*9*).

Low aqueous solubility of carotenoids is always a challenge in drug development. Although these phytochemicals have shown remarkable efficacy in cell line studies, they have shown limited efficacy in various human clinical studies. The limited oral bioavailability and rapid elimination, or combination of both, eventually result in poor therapeutic benefits (*10*). A novel drug delivery system that modifies the pharmacokinetics of existing active pharmaceutical ingredients (drug substances), such as liposomes, niosomes, nanoparticles, cyclodextrin complexes, could be used to enhance the aqueous solubility and bioavailability of chemopreventive agents. Developing a new drug delivery system is essential for targeted delivery and enhancing maximum absorption of the approved and newly investigated drug substances (*11*). Capsanthin micelle was prepared using diosgenin polyethylene glycol succinate (DPGS-1000) and characterized by various techniques. A non-ionic, amphiphilic DPGS-1000 has a hydrophobic head and hydrophilic tail with a hydrophile-lipophile balance (HLB) value of 14.2. Various spectroscopic techniques characterized the synthesized cap-DPGS-1000.

The present study aims to compare the possible cytotoxic effects of a novel keto-carotenoid capsanthin (hydrophobic) and capsanthin-loaded micelles (hydrophilic) on cell apoptosis of human triple-negative breast cancer cell line (MDA-MB-231).

## MATERIALS AND METHODS

### Chemicals

Diosgenin polyethylene glycol succinate 1000 (DPGS-1000) was provided by Phytosol India Pvt Ltd., Bangalore, India. MDA-MB-231, the triple-negative breast cancer cells were purchased from the NCCS (National Centre for Cell Science, Pune, India). The capsanthin reference standard was procured from Sigma-Aldrich, Merck (St. Louis, MO, USA). Dulbecco's modified Eagle’s medium, acridine orange, ethidium bromide, propidium iodide, Hoechst 33342, rhodamine 123, dichlorodihydrofluorescein diacetate, MTT (3-(4,5-dimethyl-thiazol-2-yl)-2,5-diphenyltetrazolium bromide), foetal bovine serum (FBS) and phosphate-buffered saline (PBS) were all procured from Merck Lifesciences, Bangalore, India.

### Extraction of capsanthin

The dried *Capsicum annuum* fruits were extracted with *n*-hexane at a temperature ranging from 40 to 60 °C (*12*). The *n*-hexane layer was filtered through a 0.5-µm filter cloth and concentrated under a vacuum (0.05 mPa).

The obtained oleoresin was further enriched by supercritical fluid extraction (40–60 °C and 25–50 mPa). The enriched extract was saponified using 50% alcoholic potassium hydroxide at 80–85 °C for 2 h. The alcohol was removed under vacuum, and the product was washed with hot water until neutral pH. Ethyl acetate was used to extract the saponified capsanthin, and water-soluble impurities were removed by washing with water. Anhydrous sodium sulfate was used to dry the ethyl acetate and capsanthin extract was concentrated under a vacuum (*13*).

### Preparation and characterization of cap-DPGS-1000

The cap-DPGS-1000 was prepared by the hot-melt extrusion method (*14*). First, a three-neck round-bottom flask fitted with an overhead stirrer was kept in an oil bath and heated to 120–130 °C. A mass of 50 g DPGS-1000 was added to the flask, and 50 g capsanthin extract were added slowly under stirring. In the mixture, the molar ratio of DPGS-1000 to capsanthin extract was 1:1. After adding the capsanthin extract, the stirring was continued, and the same temperature was maintained for two more hours for complete dissolution. When cooled, 500 mL distilled water were added, the solution was filtered and concentrated under vacuum.

UV-visible spectroscopy characterization and HPLC quantification of cap-DPGS-1000 were done as per the method described in FAO JECFA monograph (*15*). The UV-Vis spectrum was recorded on a spectrophotometer (model 1900i; Shimadzu Corporation, Kyoto, Japan) by dissolving the samples in acetone. The HPLC quantification was performed with the Shimadzu i-Series Plus HPLC (model LC-2030C; Shimadzu Corporation) using binary gradient elution. Reversed-phase column L1 loaded according to United States Pharmacopeia (USP) (SunFire, Waters Corporation, Milford, CT, USA) with a dimension of 250 mm×4.6 mm and particle size of 5 µm was used. The capsanthin reference standard from Sigma-Aldrich, Merck was used for the identification and quantification. Acetone (solvent A) and water (solvent B) were used as mobile phases. The gradient program described in the FAO monograph was followed (*15*). The capsanthin reference standard (0.01 mg/mL) and capsanthin extract (0.5 mg/mL) were dissolved in acetone and cap-DPGS-1000 (1 mg/mL) was dissolved in water. The solutions were filtered through a 0.45-µm filter before injection.

The total flow rate was maintained at 1.2 mL/min, and the detection wavelength was set at 474 nm. Capsanthin in the capsanthin extract and cap-DPGS-1000 was identified based on the retention time of the capsanthin reference standard.

### Particle size analysis

The mean particle diameter of cap-DPGS-1000 and capsanthin extract was measured by phase analysis light scattering performed on particle size analyzer (Brookhaven Instruments, Holtsville, NY, USA).

The samples were dispersed in deionized and distilled water followed by sonication for 5 min at room temperature. A scanning electron micrograph (SEM) of the aqueous dispersion was recorded on a scanning electron microscope (model EVO 18; Carl Zeiss, White Plains, NY, USA) by spreading the micelle dispersion over a carbon tape and drying it under a nitrogen stream.

### Fourier-transform infrared analysis

Attenuated total reflectance Fourier-transform infrared (FTIR) spectra of capsanthin extract and cap-DPGS-1000 were analyzed to confirm any change in the structure and functional groups after capsanthin loading using an FTIR spectrophotometer (model Nicolet iS5; Thermo Fisher Scientific, Waltham, MA, USA). The wavenumber range was set at *ṽ*=400–4000 cm^−1^.

### X-ray diffraction analysis

The crystallographic nature of capsanthin extract and cap-DPGS-1000 was evaluated by X-ray diffraction (XRD). Using a multipurpose diffractometer (model X‘Pert Pro; Philips, Farnborough, UK), the test items were scanned at a voltage of 40 kW and 30 mA. The scanning rate was 3 °C per min, and the angle was maintained at 2*θ/*°.

### In vitro drug release studies

The cumulative drug release of cap-DPGS-1000 was studied on a calibrated dissolution apparatus (model DS 8000; Labindia Analytical Instruments Pvt. Ltd., Thane, India) with paddle stirring speed 100 rpm, and the bath temperature was set at (37±0.5) °C with a dissolution volume 900 mL. A drug release experiment was conducted using 500 mg cap-DPGs-1000 in each bowl. The drug was dissolved for the first 2 h in simulated gastric fluid (buffer at pH=1.2) followed by 6 h in simulated intestinal fluid (buffer at pH=6.8). The buffer at pH=1.2 was prepared by mixing 250 mL of 0.2 M potassium chloride solution with 425 mL of 0.2 M HCl and diluted to 1000 mL with water.

The phosphate buffer at pH=6.8 was prepared by mixing 200 mL of 0.2 M potassium phosphate monobasic with 89.6 mL of 0.2 M sodium hydroxide, diluted to 1000 mL with water. At predetermined intervals, 5 mL of dissolution medium was removed for quantification and replaced with the same volumes of similar medium (*16*). The release profile quantification of capsanthin was determined by the WHO HPLC method (*15*). All measurements were performed in triplicate.

### Cell cultivation

MDA-MB-231 triple-negative breast cancer cells were purchased from the National Centre for Cell Science, Pune, India. The cells were cultured in Dulbecco’s modified Eagle’s medium (DMEM) supplemented with foetal bovine serum (*φ*(FBS)=10%) and 1% (*m*/*V*) antibiotics (penicillin/streptomycin) at 37 °C in a humidified atmosphere with 5% CO_2_ and 95% oxygen (*17*).

### In vitro cytotoxicity assay

MTT (3-(4,5-dimethyl-thiazol-2-yl)-2,5-diphenyltetrazolium bromide) assay was performed to assess the cytotoxicity of capsanthin extract and cap-DPGS-1000 against human triple-negative cell breast cancer cells. MDA-MB-231 cells were seeded at a density of 10^4^ cells per well in 96-well plates and incubated overnight. The next day the cells were treated with different concentrations of *γ*(capsanthin extract)=0.5, 1, 2, 4, 6, 8, 10, 25, 50, 75 and 100 μg/mL and *γ*(cap-DPGS-1000)=0.5, 1, 2, 4, 6 and 8 μg/mL for 24 h. After completing the incubation time, 20 μL of freshly prepared MTT (5 mg/mL in PBS) were added to each well, followed by incubation in a CO_2_ incubator (Thermo Fisher Scientific) for 3 h. The medium was removed thoroughly, and 200 μL of dimethyl sulphoxide were added to each well to dissolve the formazan crystals. Finally, the absorbance of the 96-well plates was read at 590 nm using a microplate reader (Bio-Rad Laboratories, Hercules, CA, USA).

The relative cell viability (in %) after treatment with capsanthin extract and cap-DPGS-1000 was normalized with control. The cell viability was calculated using the following equation:



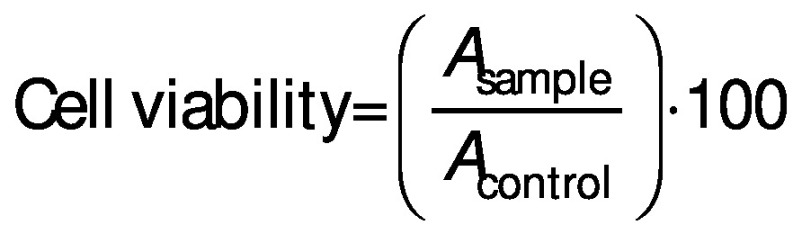



### Morphological staining

The morphology and membrane permeability of MDA-MB-231 cells treated with capsanthin extract and cap-DPGS-1000 were analyzed using the acridine orange/ethidium bromide (AO-EB) dual staining method (*18*). In brief, MDA-MB-231 cells (10^5^ cells per well) were grown in 6-well plates and incubated with half maximal inhibitory concentration (IC_50_) of capsanthin extract and cap-DPGS-1000 for 24 h.

The medium was removed after incubation, and the cells were washed with PBS and stained with a 2-μL mixture of AO/EB (100 μg/mL) for 5 min. The cells were visualized under a fluorescence microscope (model EX310; ACCU-SCOPE, Commack, NY, USA) at 20× magnifications.

### Detection of apoptotic cells

The cell-permeant nuclear stain Hoechst 33342 (*19*) and propidium iodide (PI) (*20*) were used to identify the apoptotic cells. MDA-MB-231 cells (10^5^ cells per well) were cultured and treated with IC_50_ value of capsanthin extract and cap-DPGS-1000 for 24 h. The media were removed, and sufficient Hoechst 33342 stain (10 mg/mL) and PI (10 g/mL) were added separately to cover the cells. After incubation for 10–12 min in the dark, the media were removed, washed with PBS twice, and visualized under a fluorescence microscope (model EX310; ACCU-SCOPE) at 20× magnification.

### Evaluation of mitochondrial membrane potential

Rhodamine 123 (Rho 123) staining was used to evaluate mitochondrial membrane potential. The fluorescent Rho 123 dye binds to metabolically active mitochondria. MDA-MB-231 cells were grown and treated with IC_50_ value of capsanthin extract and cap-DPGS-1000 for 24 h. The cells (10^5^ cells per well) were washed with PBS, stained with Rho 123 for 1 h in the dark at 37 °C, and visualized under a fluorescence microscope (model EX310; ACCU-SCOPE) at 20× magnification.

### Generation of reactive oxygen species

The intracellular reactive oxygen species (ROS; free radicals) accumulation within MDA-MB-231 cells was analyzed by 2′,7′dichlorodihydrofluorescein diacetate (DCF-DA) staining. For this, 10^5^ cells per well were seeded into 6-well plates (grown overnight) and incubated with IC_50_ value of capsanthin extract and cap-DPGS-1000 for 24 h. The medium was removed, washed with PBS and stained with 100 μL DCF-DA (50 μM) for 25–20 min in the dark at room temperature. Fluorescence was detected using a fluorescence microscope (model EX310; ACCU-SCOPE) at 20× magnifications.

### Statistical analysis

Data normality was determined with the student’s *t*-test using GraphPad Prism v. 7.04 software (*21*). Values are given as mean±standard deviation (S.D.) obtained from three independent experiments. Statistical comparison between the treated and control groups was carried out by one-way analysis of variance (ANOVA), and p<0.05 was considered to indicate a statistically significant difference.

## RESULTS AND DISCUSSION

In women, breast cancer is the most common cancer, followed by skin cancer, and the second leading cause of death (*22*). Each year, approx. one million women join the breast cancer battle (*23*). Cancer pathogenesis involves several etiological factors, including age, family history, unbalanced diet and lack of physical activity, and endocrine factors, such as hormonal imbalance (*24*). In a multicellular organism, programmed cell death is essential for normal growth, known as apoptosis, but this process may be blocked in cancer cells. The uncontrolled cell division and accumulation of abnormal cells are due to induction to apoptosis (*25*). Therefore, apoptosis plays a significant role in cancer treatment (*26*).

Exploring complementary and alternative breast cancer treatment options is inevitable due to the irreversible and undesirable effects associated with chemotherapy (*27*, *28*). Secondary metabolites from plants have been extensively researched for their biological effect, especially inducing apoptosis in breast cancer (*29*, *30*). Naturally available phytochemicals play an essential role in treating several pathologies, among them cancer (*31*). Epidemiological studies and experimental results suggested that carotenoids can be used as dietary anticancer agents. The free radical quenching and neutralizing ability of carotenoids can exert antiproliferative and antitumour activities (*32*). Carotenoids reduce inflammation that can stimulate breast cancer tumour growth (*33*). When combined, carotenoids can be used to suppress breast cancer cell growth. Capsanthin significantly induced apoptosis and reduced cell viability in MCF-7 cells dose-dependently (*34*). Capsanthin causes oxidative stress and decreased mitochondrial membrane potential by reducing glutathione and catalase in MCF-7 cells.

### Preparation and characterization of capsanthin-loaded micelles

**Fig. S1:**
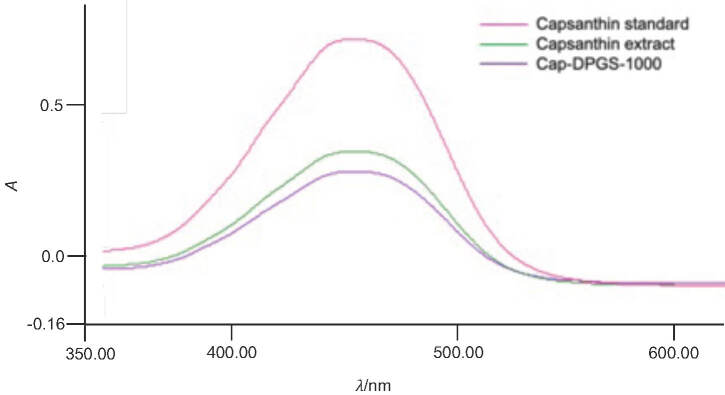
UV-Vis absorbance spectra of: capsanthin standard, capsanthin extract and capsanthin-loaded micelles of diosgenin polyethylene glycol succinate 1000 (cap-DPGS-1000)

**Fig. S2:**
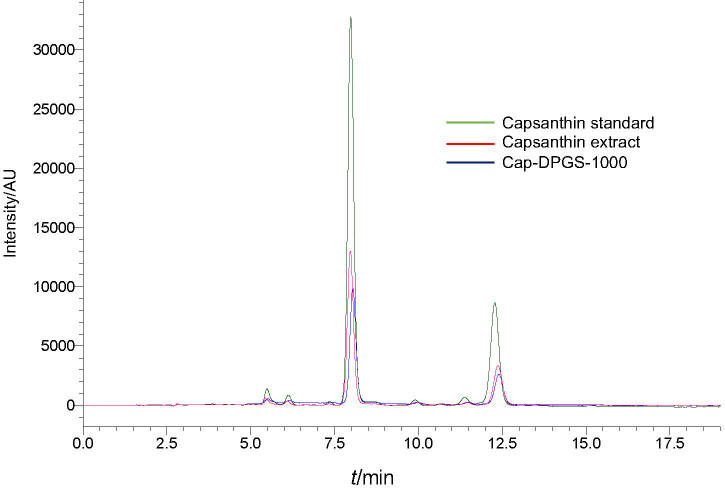
HPLC chromatograms of: capsanthin standard, capsanthin extract and capsanthin-loaded micelles of diosgenin polyethylene glycol succinate 1000 (cap-DPGS-1000)

**Fig. S3:**
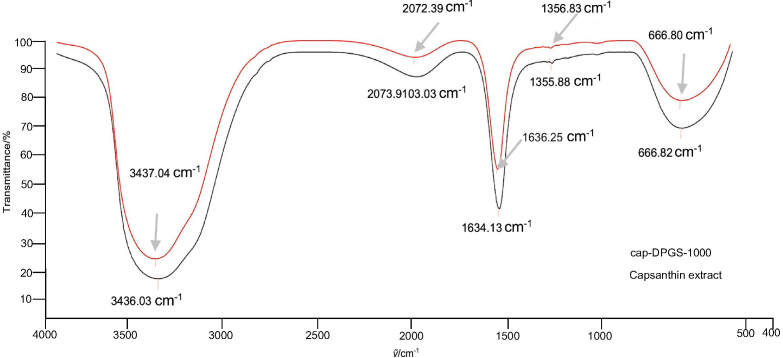
FTIR spectra of: capsanthin extract and cap-DPGS-1000

**Fig. S4:**
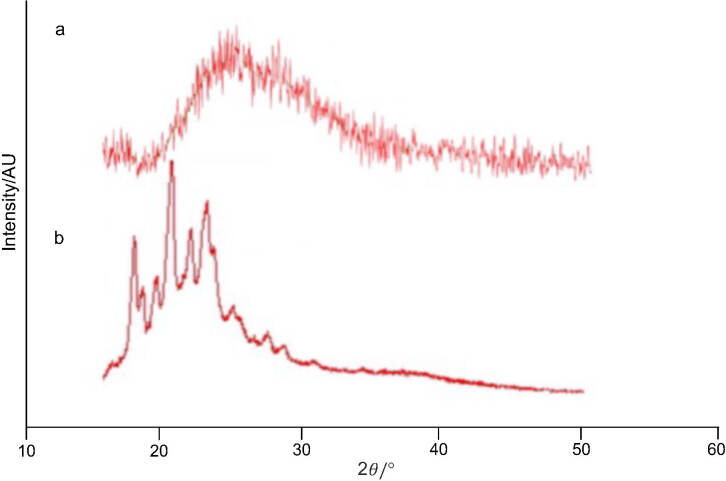
XRD spectra of: cap-DPGS-1000 (a), and capsanthin extract (b)

Capsanthin was successfully loaded into diosgenin polyethylene glycol succinate by solid dispersion technique. The mass fraction of capsanthin in capsanthin extract and cap-DPGS-1000 was >50 and >20, respectively. Furthermore, UV-Vis spectrum (Fig. S1) and HPLC characterization (Fig. S2) confirmed the presence of capsanthin in cap-DPGS-1000. The changes in the structure and functional groups were further investigated by FTIR after the encapsulation of capsanthin. The FTIR spectra of capsanthin extract exhibit a -CH_3_ bending band at 1365 cm^-1^, a stretching band at 1634 cm^-1^, typical for conjugated ketone (-C=0), and a broad  -OH stretching band at 3436 cm^-1^ (*35*). The IR spectra of cap-DPGS-1000 are concordant with capsanthin extract. The FTIR spectra shown in Fig. S3 suggest that no change in the structure or the functional group due to encapsulation of capsanthin in the micelles occurs. The crystalline nature of capsanthin extract and cap-DPGS-1000 was further analyzed by XRD (Fig. S4). The crystalline structure of capsanthin in the capsanthin extract was observed as distinct peaks in the range of 15°≤2*θ*≥30°. However, in capsanthin-loaded micelles, no such crystalline peaks were observed. The disappearance of characteristic crystalline peaks in the cap-DPGS-1000 demonstrated that capsanthin molecules exist as amorphous or disordered crystalline forms. Thus, the encapsulation of capsanthin in DPGS-1000 was further confirmed by the XRD pattern. The solubility of a material is higher when it is in the amorphous form than the more stable crystalline form because of the higher Gibbs free energy. Most recently, amorphous solid dispersion has been one of the most significant strategies to enhance the bioavailability of poorly aqueous soluble drugs (*36*). The higher solubility and more significant biological effect of cap-DPGS-1000 than of capsanthin extract are due to lack of crystalline nature.

### Investigation of physicochemical properties of capsanthin-loaded micelles

Particle size is an important factor for insoluble drugs (*37*) and hydrophobic phytochemicals. Smaller particles have a large surface area, higher dissolution and faster absorption than the bigger particles. The dynamic light scattering (DLS) technique was used to examine the particle size distribution and zeta potential of capsanthin-loaded micelles. The average particle size of the micelles was *d*=(508±3) nm, almost 80 times lower than of the capsanthin extract (*d*=(31993±5) nm). Particle size and their distributions in a sample can be defined by polydispersity index (PDI).

The value of PDI ranges from 0.0 (uniform particle size) and 1.0 (multiple particle size). Usually, the acceptable value for a micelle is 0.2 and below. From the PDI value for cap-DPGS-1000 (0.3±1.1), we can conclude that the particle size is almost uniform, but for the free capsanthin, the PDI is 0.6±0.9, which is a characteristic of multiple particle size groups (*38*). The zeta potential of micelles is one of the most critical factors when nanoparticles enter the bloodstream. The positive charge of micelles leads to an increase in the removal of nanoparticles by the reticuloendothelial system and increases adsorption by non-specific proteins (*39*, *40*). The negative zeta potential of the capsanthin-loaded micelles was found to be (-38.5±0.3) mV, which indicates greater stability of the particles in the solution. The data are shown in Table 1. The size and morphology of the cap-DPGS-1000 were confirmed by SEM imaging.

**Table 1:** Average particle size, zeta potential and polydispersity index (PDI) of capsanthin extract and capsanthin loaded micelles (cap-DPGS-1000)

Extract	*d̅*(particle)/nm	®*ξ*/mV	PDI
Capsanthin extract	(31993±5)	(-33.2±1.8)	(0.6±0.9)
Cap-DPGS-1000	(508±3)	(-38.5±2.7)	(0.3±1.1)

Data are expressed as mean value±standard deviation (*N*=3). DPGS-1000=diosgenin polyethylene glycol succinate 1000

**Fig 1:**
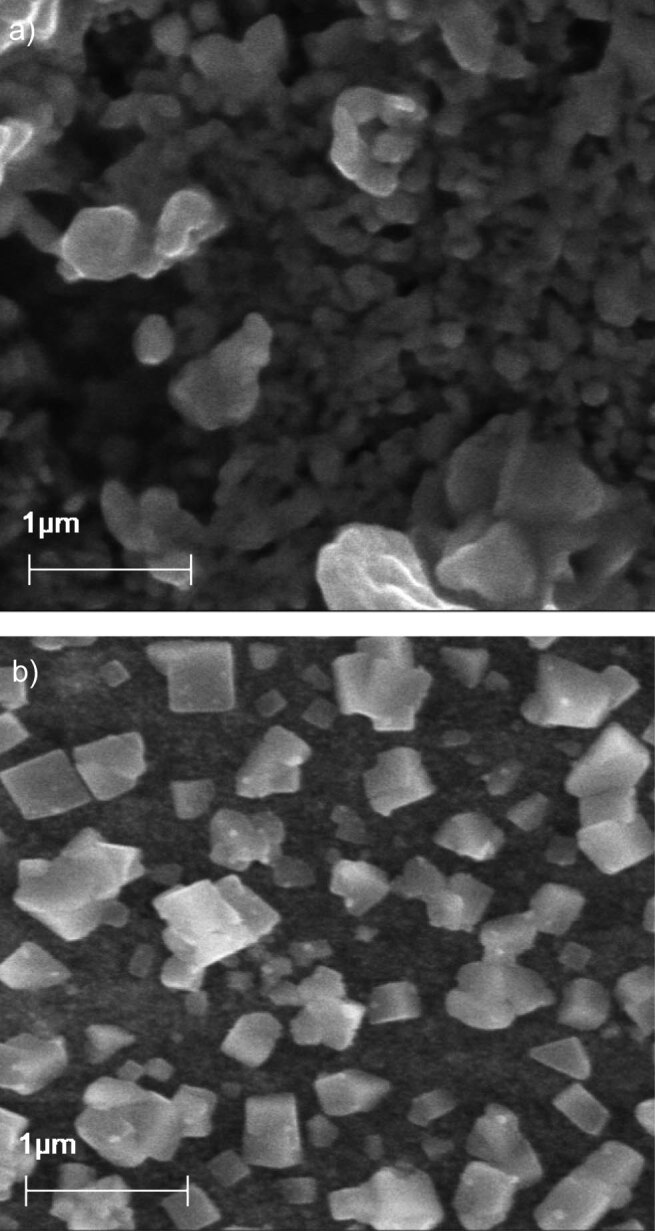
Scanning electron microscopy (SEM) micrographs of: a) capsanthin extract, and b) capsanthin-loaded micelles of diosgenin polyethylene glycol succinate 1000 (cap-DPGS-1000). Magnification=30K×, electron high tension (EHT)=15 kV, working distance (WD)=9.0 mm, signal A=secondary electrons SE1

The capsanthin and cap-DPGS-1000 were dispersed in water, and SEM readings were recorded (Fig. 1). Capsanthin-loaded micelles show that the particles have an average size of 50–100 nm, which is well within the optimal range below 200 nm. There is a notable decrease in the size between the capsanthin extract and the cap-DPGS-1000, probably due to DPGS-1000 preventing aggregation. Particle size reduction enhances drug wettability and bioavailability significantly. Lipophilic drugs suffer from slow dissolution, incomplete release and poor bioavailability. The poor solubility and bioavailability of lipophilic capsanthin can be enhanced by reducing the particle size by converting it into capsanthin-loaded micelles.

### Water solubility and drug release

**Fig. S5:**
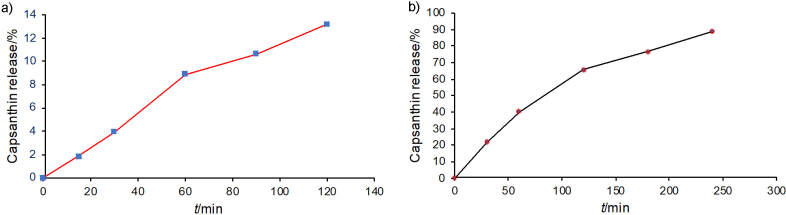
Release of capsanthin with time from capsanthin-loaded micelles of diosgenin polyethylene glycol succinate 1000 (cap-DPGS-1000) in: a) simulated gastric buffer (pH=1.2) and b) simulated intestine buffer (pH=6.8). Data represent the mean values±standard deviation (*N*=3). Dissolution conditions: *V*(medium)=900 mL, 100 rpm paddle stirring speed, method=USP type II

Water solubility is an essential parameter for drug absorption, desired concentration in the systemic circulation, bioavailability and a desired biological response (*41*). The amphiphilic cap-DPGS-1000 can form self-assembled micelles in aqueous solutions. Capsanthin remains as a hydrophobic core, while DPGS-1000 can form a hydrophilic outer shell of the micelles. Cap-DPGS-1000 micelles were dissolved in simulated gastric fluid (buffer at pH=1.2) and it was found that a small amount of capsanthin was released (Fig. S5a). The cap-DPGS-1000 particles were intact even after 120 min, and the acid-resistant property ensured that very low amount of capsanthin (13.19%) was released in simulated gastric pH. The release profiles of capsanthin from cap-DPGS-1000 for 240 min in simulated intestinal fluid (pH=6.8) are shown in Fig. S5b). From this profile, it was found that the capsanthin was gradually released over time, and after 240 min 88.82% was released. Cap-DPGS-1000 did not show any rapid dissolution, probably due to capsanthin being strongly bound or adsorbed in cap-DPGS-1000 (*42*). Based on the above results, the formulation method affects the dissolution profile, and capsanthin loaded by the solid dispersion method can influence rapid dissolution and delayed release characteristics (*43*).

### In vitro cytotoxicity and apoptosis

Programmed cell death (apoptosis) is crucial for normal cell development and turnover, but the apoptosis pathway is deregulated in cancer. Triple-negative breast cancer is defined by the lack of estrogen and progesterone receptor expression and HER2 (human epidermal growth factor receptor 2) overexpression/amplification, and represents 10-15% of all breast cancer patients (*44*). Carotenoids may exhibit cytotoxic activity in various cancer cell types (*45*). Lutein, the extensively studied carotenoid, inhibits a variety of human breast cancer cells, including the BT-474 (ER/PR^+^HER2^+^), MDA-MB-453 (triple-negative), and MDA-MB-231 (triple-negative) cell lines. Furthermore, lutein significantly reduced colony numbers in MCF-7 and MDA-MB-468 cells (*46*). Lycopene, zeaxanthin and capsanthin showed the highest total apoptosis in the MDA-MB-231 breast cancer cell line, according to Molnár *et al.* (*47*).

**Fig. 2:**
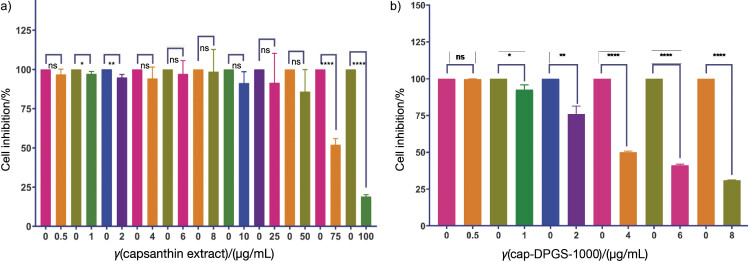
Tetrazolium dye MTT assay of: a) capsanthin extract, and b) capsanthin-loaded micelles of diosgenin polyethylene glycol succinate 1000 (cap-DPGS-1000) for 24 h (0=control). The values are presented as the mean±standard deviation (*N*=3). *p<0.05, **p<0.01, ****p<0.0001, ns=not significant

**Fig. 3:**
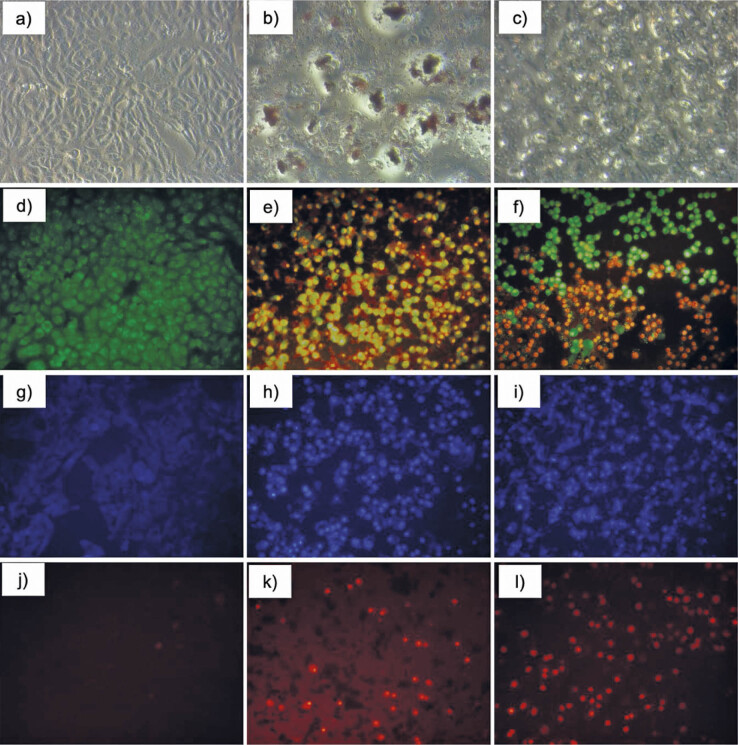
Morphology of MDA-MB-231 cells after treatment with IC_50_ of capsanthin extract or capsanthin-loaded micelles of diosgenin polyethylene glycol succinate 1000 (cap-DPGS-1000): a−c) control and inverted-phase contrast images after treatment with capsanthin extract and cap-DPGS-1000, respectively, d−f) control and AO-EB staining images after treatment with capsanthin extract and cap-DPGS-1000, respectively, g−i) control and Hoest-33342 staining images after treatment with capsanthin extract and cap-DPGS-1000, respectively, j−l) control and propidium iodide staining images after treatment with capsanthin extract and cap-DPGS-1000, respectively. Control=stained untreated MDA-MB-231 cells

The results of these MTT assays on the MDA-MB-231 cancer cell line, show that the percentages of viable cells in the groups treated with different concentrations of capsanthin and capsanthin-loaded micelles were significantly reduced in a dose-dependent manner, and the reduction was statistically significant compared with the control group (p<0.05). The IC_50_ at 24 h for capsanthin and capsanthin-loaded micelles were (81.1±1.5) and (3.1±1.1) μg/mL, respectively (Fig. 2). Our study validates the previous findings of the cytotoxic activity of capsanthin, but capsanthin-loaded micelles against MDA-MB-231 were not studied and reported earlier. The conspicuous morphological changes such as growth inhibition, distorted cell shape and cytoplasmic condensation were observed by inverted phase-contrast microscope, compared to the untreated control (Fig. 3a), in capsanthin extract (Fig. 3b) and cap-DPGS-1000 treated-cells (Fig. 3c). The cells in the control group were viable and uniform in shape. The morphological alterations induced by capsanthin extract and cap-DPGS-1000 were observed using acridine orange-ethidium bromide (AO-EB) dual staining (nucleic acid binding stains). Under a fluorescence microscope, untreated cells appeared uniformly shaped, of light green colour and without distortion. The morphology of treated cells was entirely different from the untreated ones. The early process of apoptosis, such as nucleus condensation and cell shrinkage (*48*), were observed in the treated cells. The treated cells were smaller due to the dense cytoplasm and more tightly packed organelles. The AO-EB staining of control, cells treated with IC_50_ value of capsanthin extract and cap-DPGS-1000, and change in the morphology are shown in Figs. 3d, 3e and 3f, respectively.

The occurrence of apoptosis was further confirmed by the DNA-binding dye Hoechst 33342, which detects chromatin condensation, a hallmark of apoptotic cell death (*49*), while propidium iodide (PI) staining was used to confirm AO-EB staining results.

These nucleus-binding dyes stain all the cells without discriminating their viability (*50*). The nuclei of capsanthin extract and cap-DPGS-1000-treated cells showed a brighter blue fluorescence than the untreated cells. They were fragmented and shrank compared to the untreated control group. The Hoechst 33342 control, cells treated with IC_50_ value of capsanthin extract, and cap-DPGS-1000 are shown in Figs. 3g, 3h and 3i, respectively.

Furthermore, the apoptosis and morphological changes in the capsanthin extract and cap-DPGS-1000-treated cells were confirmed by red PI staining, which can stain necrotic and late apoptotic cells. Distinguishable changes like condensed nuclei and apoptotic bodies were observed in the treated cells compared to control. The fluorescence image of PI-stained control, cells treated with IC_50_ value of capsanthin extract and cap-DPGS-1000 are shown in Figs. 3j, 3k and 3l, respectively.

### Mitochondrial membrane potential

**Fig. 4:**
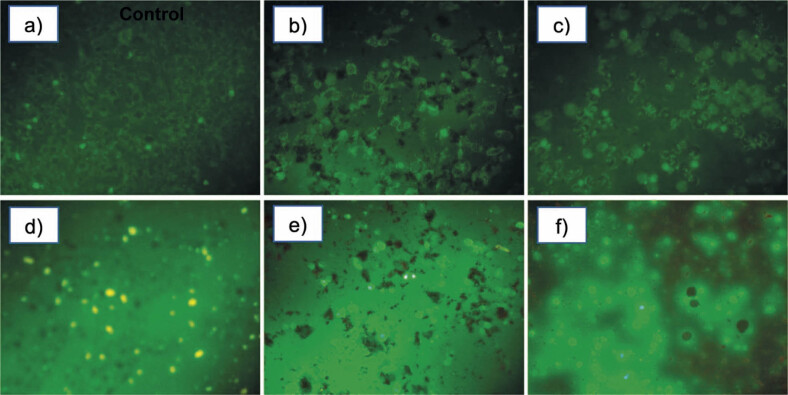
Morphology of MDA-MB-231 cells after treatment with IC_50_ of capsanthin extract or capsanthin-loaded micelles of diosgenin polyethylene glycol succinate 1000 (cap-DPGS-1000): a−c) control and rhodamine staining images after treatment with capsanthin extract and cap-DPGS-1000, respectively, d−f) control and 2′,7′-dichlorofluorescein diacetate staining images after treatment with capsanthin extract and cap-DPGS-1000, respectively. Control=stained untreated MDA-MB-231 cells

Mitochondrial membrane potential was evaluated after treatment of MDA-MB-231 cells with IC_50_ value of capsanthin extract and cap-DPGS-1000 for 24 h, and Rho 123 staining. The control cells with high membrane potential showed green fluorescence under a fluorescence microscope (Fig. 4a). The decrease in the fluorescence in treated cell lines indicates the decrease in the mitochondrial membrane potential, as shown in Figs. 4b and 4c.

### Evaluation of intracellular reactive oxygen species generation

It is well known that reactive oxygen species (ROS) generation is usually associated with cell apoptosis (*51*). Generation and accumulation of ROS can elicit apoptosis and inhibit the cell cycle, aggravating oxidative stress in cancer cells (*52*). To compare the level of ROS generation in MDA-MB-231 cells with or without capsanthin extract and Cap-DPGS-1000, the fluorescent dye, DCF-DA (2′,7′-dichlorofluorescein diacetate) stain was used and the cells were examined under a fluorescence microscope, showing a significant increase in the fluorescence (Figs. 4e and 4f) compared to control (Fig. 4d).

## CONCLUSIONS

In this study, we have extracted capsanthin from sweet red pepper (*Capsicum annuum*) fruit and developed capsanthin-loaded diosgenin polyethylene glycol succinate 1000 (cap-DPGS-1000) micelles successfully the first time, and the micelles exhibited suitable physicochemical properties. A solid dispersion technique was employed to prepare the cap-DPGS-1000 micelles as a delivery system for the hydrophobic capsanthin. The FTIR and other characterization data confirmed no changes in the capsanthin structure and functional groups in the capsanthin-loaded micelles. The capsanthin-loaded micelles were nanosized with low polydispersity, indicating the size uniformity. *In vitro* release behaviour of capsanthin was controlled for 240 min. Finally, capsanthin extract and capsanthin-loaded micelles were evaluated for their cytotoxic activity on triple-negative breast cancer cells MDA-MB-231. The results of the study show that both capsanthin extract and cap-DPGS-1000 had cytotoxic effects on MDA-MB-231 breast cancer cells. The IC_50_ value of the cap-DPGS-1000 micelles against MDA-MB-231 breast cancer cells was 3.1 μg/mL, which is much lower than of capsanthin extract (IC_50_=81.1 μg/mL). Capsanthin extract and capsanthin-loaded micelles are promising drug candidates to induce apoptosis and increase reactive oxygen species in cancer cells.
